# Mild Cognitive Impairment in Rural Areas: Research Advances and Implications for Clinical Practice and Healthcare Policy

**DOI:** 10.3390/healthcare10071340

**Published:** 2022-07-19

**Authors:** Vaios Peritogiannis, Aglaia Roganaki, Eleftheria Siarava, Maria Samakouri

**Affiliations:** 1 Mobile Mental Health Unit of the Prefectures of Ioannina and Thesprotia, Society for the Promotion of Mental Health in Epirus, 54 Pashidi Str., 44445 Ioannina, Greece; 2 Department of Psychiatry, Medical School, Democritus University of Thrace, 68100 Alexandroupolis, Greece; liaroganaki@hotmail.com (A.R.); msamakou@med.duth.gr (M.S.); 3 Mouzaki Health Center, 43060 Mouzaki, Greece; 4 Department of Neurology, University of Ioannina, 45500 Ioannina, Greece; esiarava@hotmail.com

**Keywords:** mild cognitive impairment, mild neurocognitive disorder, rural areas, education, social participation

## Abstract

Mild cognitive or neurocognitive impairment (MCI) may be more prevalent in rural areas. Differences between rural and urban MCI patients in terms of risk factors, course and prognosis are rarely reported. The present review aims to summarize the latest research on MCI in rural areas. A literature search was performed in the databases of PubMed, Scopus and ScienceDirect for articles published over the last decade. Eleven articles were included in this review, reporting on the differences between rural and urban MCI patients. Several risk factors, such as older age, lack of activities and food insecurity have been associated with MCI in both rural and urban areas, whereas others, such as obesity, adverse childhood experiences and plasma chemokine C-C motif ligand 11 (considered as a potential negative regulator of neurogenesis), differed according to the place of residence. No specific protective factor for rural women has been reported. There is some evidence that MCI may present earlier in rural residents, but that progression to dementia may be more rapid in urban residents. It seems that there may be clinically relevant differences in the onset, course and prognosis of MCI with regards to the place of residence (urban vs rural). Those differences should be taken into account for the design of health policies and service delivery across different settings.

## 1. Introduction

Mild cognitive impairment (MCI), or mild neurocognitive disorder, according to the latest classification of the American Psychiatric Association (DSM-5), is a condition that is characterized by cognitive decline with minimal impairment of instrumental activities of daily living of the individual and the preservation of baseline functioning [1,2]. Large population-based studies have shown that the overall prevalence of MCI in the elderly may be up to 12.5%. Hypertension and stroke have been found to be significant risk factors for MCI, whereas higher educational levels and active social engagement are significant protective factors [3]. MCI is widely considered to be the intermediate stage of cognitive impairment between the changes seen in normal cognitive aging and those associated with dementia. Progression to dementia related to Alzheimer’s disease is the most common outcome of MCI, although potentially reversible causes, such as metabolic or systemic, should be taken into account [4]. Although persons with MCI are at higher risk of progressing to dementia than age-matched controls, in several cases cognitive function may remain stable, or even return to intact [2].

Less is known regarding MCI in the rural context. It has been suggested that despite improved access to health services, inadequate diagnosis and management of dementia may be still common, particularly in rural areas [5]. According to recent research, receiving an early diagnosis of cognitive decline may be difficult in rural areas due to the limited access to assessments [6]. In an earlier population-based study in northern Portugal, it was found that the prevalence of cognitive impairment with or without dementia in rural areas was much higher than in urban locations. Cerebrovascular disease and other vascular factors accounted for almost half of the overall cognitive impairment, whereas other medical and neuropsychiatric comorbidities and low levels of education had been also associated with cognitive impairment [7]. A more recent systematic review of Chinese studies confirmed that the prevalence of MCI in rural areas was higher than the corresponding prevalence in urban ones [8]. Such differences in cognitive function between rural and urban Chinese older residents were very recently found to be mediated by social participation [9].

These notions make the study of MCI in the rural context both timely and relevant. The aim of the present review was therefore to present an up-to-date account of the study of MCI in rural areas, with a focus on the differences between rural and urban patients; and to stress the importance of studying MCI in rural areas for clinical practice and healthcare policy.

## 2. Materials and Methods

A review of the literature was conducted. The search was run in the PubMed, Scopus and ScienceDirect databases with the use of the key-words “mild cognitive impairment” or “mild neurocognitive impairment” and “rural areas”, with cross-referencing across the databases. It has been argued that PubMed is the optimal tool in biomedical electronic research, whereas Scopus covers a wider journal range and offers the opportunity for citation analysis [10]. Accordingly, it was hypothesized that all of the relevant articles would have been cited in these three databases. Mild cognitive or neurocognitive impairment were defined according to the contemporary diagnostic criteria (*International Classification of Disease, 10th revision* and *Diagnostic and Statistical Manual for Mental Disorders-fifth edition*, respectively). However, the precise definition of “rural” is still a limitation in the literature [11]; that is, there is no standardized definition of rural areas in research, and measurements vary across countries and studies [12]. For the purposes of this review, rural was considered self-defined, as conceptualized by the authors of the studies. The search was limited to original articles published over the last decade (2012–2022). All types of research design were included as long as studies had used a control group of urban patients and reported on the differences among rural/urban residents with MCI. Articles were considered regardless of the specific objectives of the studies, for example, outcomes, treatment aspects, etc. The references of the originally retrieved articles were also searched for additional articles. We excluded studies that addressed prevalence of MCI in rural areas, because there is a recent systematic review on the subject [8]. The main focus of the review was on patients rather than families, so studies addressing interventions on families or other caregivers were excluded. Different publications from the same study addressing different objectives were considered separately.

## 3. Results

**Figure 1:**
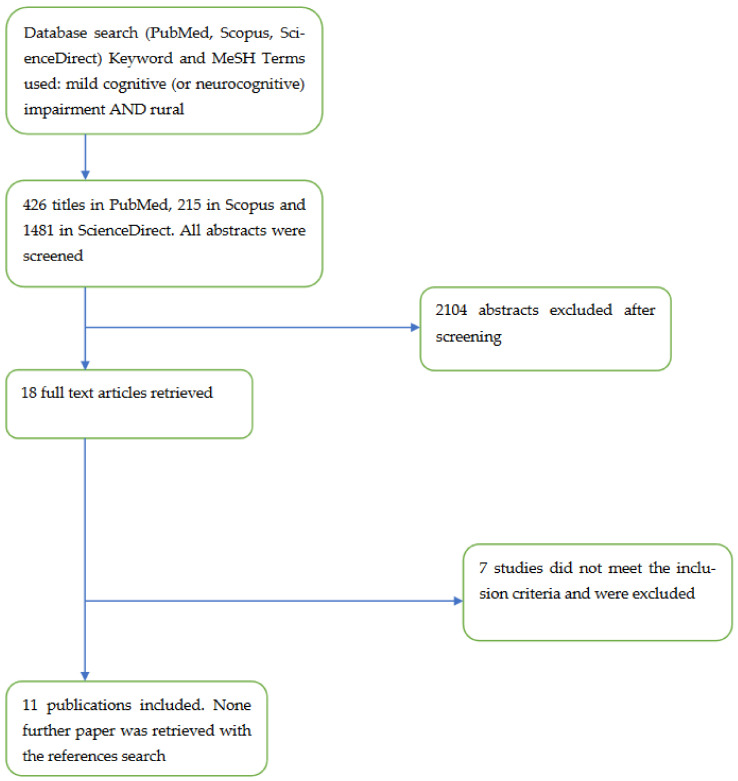
Flow chart of the screening procedure for the studies included in the present review.

The process followed for the search is illustrated in the flowchart (Figure 1). In the initial search, 426 publications in PubMed, 1481 in Science Direct and 215 in Scopus were retrieved. The titles and abstracts of all papers were screened and, after the exclusion of the irrelevant articles, 18 papers were identified as relevant. Subsequently, all relevant papers were read in full, and 7 more were excluded. No additional papers were retrieved from the references of the originally selected articles. Finally, 11 papers were included in this review, that are presented briefly in Table 1 and Table 2. For descriptive reasons, studies were divided between those that reported on the differences in risk factors for MCI (Table 1) and those that reported on its diagnosis and prognosis (Table 2). Six studies had been conducted in China, 3 in Western countries (namely the USA and France), 1 in India and 1 in Thailand.

### 3.1. Studies Reporting on Risk Factors for MCI

With regards to the risk factors of cognitive decline and MCI, a total of 8 studies were detected (Table 1). According to a large study from China [20], the identified risk factors in urban populations were older age, a lack of physical activities, a history of diabetes and having three or more children. In rural residents, the female gender, older age, exposure to pesticides, a history of encephalitis, meningitis and head trauma were identified as risk factors for MCI. A subsequent study in Thailand [19] identified as risk factors for MCI (for both urban and rural residents) the female gender, older age, a low level of education and the absence of active leisure. Moreover, in urban populations but not in rural, bad perceived health was a risk factor for MCI, whereas in rural but not in urban residents, poor financial status was a risk factor for MCI. In another large study from China [16] it was found that the same productive activities (a term that refers to older adults’ activities that produce socially valued services, regardless whether they are paid or not) were not beneficial for urban and rural male and female residents, in terms of cognitive function enhancement. Specifically, the care of grandchildren and being a volunteer were the most beneficial for urban women, whereas in the case of urban men, informal helping (that is, providing help to others living outside of the household, such as neighbors, friends and relatives) was the most beneficial. For rural men, the paid employment had been the most beneficial. With regards to rural-dwelling women, no beneficial activities were found. Interestingly, agricultural work was related to cognitive decline in both men and women living in urban areas and in men in rural areas. In a more recent study from China [15], obesity was associated with the increased risk of cognitive impairment in urban residents aged 65–69. Being overweight was associated with higher cognitive impairment in rural residents aged 80+, but not in urban residents. Furthermore, in rural areas, being underweight was related with a higher dementia risk in residents aged 80+. The most recent Chinese study aimed to explore the association between adverse childhood experiences (comprising poor socioeconomic status, food deprivation, loneliness, poor relationships with parents and abuse) and MCI in a very large sample of 11,475 adults aged ≥45 years. There were differences in the correlations of MCI between rural and urban residents. In the rural subsample of participants, poor family socioeconomic status, food deprivation, loneliness, poor family relations and adverse neighborhood environments were associated with MCI. In urban residents, only the father’s education, loneliness and poor family relations were associated with MCI [14]. The most recent study from India [13] showed that food insecurity was associated with cognitive impairment in adults older than 60 years in both rural and urban areas. However, rural residents with food insecurity were more likely to have cognitive impairment compared with urban residents with food insecurity. The authors interpreted these findings as people in rural areas potentially having more health problems, poorer cooking skills and limited food supports.

Other research attempted to address biological factors in patients with MCI in relation to their place of residence. In a study from the USA concerned with Mexican-Americans, data from two cohorts, FRONTIER for rural and HABLE for urban residents, were analyzed [18]. In both cohorts, potassium levels in fasting blood samples, were higher in people with MCI compared with cognitively normal participants. No comparison between the potassium levels of urban and rural residents was performed. A study from France [17] explored the association between cognitive impairment and the plasma chemokine C-C motif ligand 11 (CCL11), a potential negative regulator of neurogenesis. Interestingly, increased CCL11 was associated with poorer cognitive performance in people living in rural areas. This association was not found in people living in urban areas, and the authors concluded that there may be environmental factors that influence the relation between CCL11 and cognitive impairment. However, the precise mechanisms that underlie the environmental influence on CCL11 and cognition remain unknown.

### 3.2. Studies Reporting on Diagnosis and Prognosis

Three studies have been identified that addressed diagnosis and prognosis of MCI in rural elderly residents (Table 2). A study conducted in the Appalachia Alzheimer Center, USA, [23] revealed that rural residents seek diagnosis earlier following the onset of symptoms, compared with the urban residents. Rural residents reported that they had more relatives with dementia compared with urban residents, so the authors hypothesized that rural patients or their families may be able to recognize the MCI symptoms earlier. In a study from China [22], it was found that although residents in urban areas had better initial cognitive status, they had a faster rate of cognitive decline compared with the residents of rural areas. Higher education level was the only variable that explained the higher initial cognitive status in urban residents. A large longitudinal study from China by Li et al. [21] evaluated the mortality risk in persons with cognitive impairment. Higher cognitive impairment was associated with higher mortality risk in both rural and urban elderly residents; however, no difference in mortality risk between rural and urban groups with similar cognitive impairment was detected.

## 4. Discussion

The prevalence of MCI in rural areas has been reported to be as high as over 26% [24], making its study in the rural context relevant and the investigation of the factors that may be associated with cognitive decline in those areas important. This review aimed to summarize the latest research on the differences between rural- and urban-dwelling older adults with MCI. Although beyond the scope of the present review, we have to note that in all [13,14,16,19,21] but one [20] the reviewed studies that also reported on the prevalence of MCI, its prevalence was higher in rural areas. This finding is in line with previous research [8] which suggested that living in rural areas is a major risk factor for MCI [3]. The potential implications of the research findings for clinical practice and mental healthcare policy are discussed below.

### 4.1. Risk Factors for MCI: Differences between Rural and Urban Areas

Recent research addressing risk factors for MCI in rural and urban contexts has yielded interesting results. As shown in Table 1, some risk factors have been associated with MCI in both rural and urban areas [13,16,19,20], whereas there were also marked differences in several factors associated with MCI across settings [14,15,17]. The association between low BMI and cognitive impairment in rural older adults is a noticeable finding, given that the prevalence of being underweight in rural areas may be higher than in urban ones [15]. Undernutrition in rural-dwelling older adults may be a serious public health concern in certain countries, such as China [15]. Moreover, the types of productive activities that are most beneficial for cognition may vary between urban and rural residence. This finding suggests that environmental demands and resources may play a role in shaping the effects of such participation on cognitive function [16]. The association of MCI in rural-dwelling older adults with the exposure to pesticides, history of encephalitis, meningitis and head trauma [20], may be in part mediated by CCL11, which is a negative regulator of neurogenesis [17].

According to recent research, it seems that the risk for developing MCI may be mediated by environmental factors. Urbanicity and the so-called urban stress have been consistently implicated in the pathophysiology of major mental disorders, such as schizophrenia [25]. However, in the case of cognitive function in the elderly it seems that urban environments may have a protective effect. This may be partly mediated by the higher education and the more opportunities for social participation of the elderly in urban locations [16,19]. There may also be other protective components of urban environments on cognitive function that should be addressed by future research.

It should be mentioned that the female gender was not consistently reported as a risk factor for MCI in both rural and urban residents across studies [19,20]. Such inconsistencies may reflect differences in methodology and measurements across studies. Moreover, recent research did not find any protective factors against MCI in rural women, and the authors suggested that the traditional and constrained role of those women as caregivers may limit the benefits they derive from caregiving [16]. These observations should raise awareness in the study on MCI in females in rural areas to elucidate potential risk and protective factors.

### 4.2. Diagnosis, Prognosis and Mortality of People with MCI in Urban and Rural Areas

There is some evidence that people living in rural areas may be diagnosed with MCI earlier than their urban counterparts [23]. On the other hand, it has been suggested that despite improved access to health services, the inadequate diagnosis of cognitive decline may be still common in rural areas [5]. Taken together, these notions could mean that MCI may indeed present earlier in rural residents. The confirmation of this suggestion by future research would be important if we are to employ preventive interventions for cognitive decline in rural residents. Interestingly, other recent research showed that rural residents with MCI display a lower rate of cognitive decline [22] compared with MCI patients living in urban areas. This may mean that once MCI is established in urban residents, progression to dementia is hastened by factors associated with the urban environment. It has been suggested that a high population density, which is often the case in urban environments, may lead to constricted life space (defined as the extent of movement through the environment covered in daily functioning) for the elderly, which has been shown to be associated with cognitive decline [26]. That would be particularly relevant in the case of urban elderly residents with MCI, who may have a potentially faster decline rate afterwards. On the other hand, rural environments may be less constricted for elderly patients with MCI, thus the rate of cognitive decline may be delayed. Notably, despite differences in the onset and course of MCI, the all-cause mortality in patients was found to be similar in rural and urban locations in a Chinese study. According to the authors, this observation indicates the scarcity of healthcare and treatment for cognitive impairment in China [21]. This suggestion is in line with the results of a previous meta-analysis, which found that the prevalence of undetected dementia was high globally, and that the rate of under-detection was higher in China and India compared with Europe and North America [27].

### 4.3. Strengths and Limitations of the Present Review

The place of residence may play a major role in the progression and outcome of neuropsychiatric diseases [28]. The present review is a systematic approach to explore the differences between people with MCI in rural and urban areas. This is the first study that reviews the different risk factors and prognoses of MCI between rural and urban residents, beyond differences in prevalence. The present review also has some limitations. The search was limited to the last decade, and not all of the search platforms were used. It was also hypothesized that the combination of the terms mild cognitive (or neurocognitive) impairment with the broad term rural areas would be sufficient for tracing all relevant articles, so we did not use additional specific terms (such as outcome or risk factors) to enhance the search. It could be argued that this combination of key-words may not be sufficient to reveal all relevant studies. However, the MeSH terms that were used cover a broader range of definitions related to cognitive decline, thus enriching the search. Furthermore, studies in languages other than English may have been missed. Finally, more than half of the studies were conducted in China, thus limiting the generalizability of findings to other countries. More studies in different countries are needed to elucidate the impact of rural and urban environment on cognition and neurodegeneration.

### 4.4. Limitations of Research

Recent findings regarding the differences in MCI between rural and urban residents may not be generalized across countries and settings due to differences in methodology and cognitive assessment of participants. Not all studies have used the age limit of 65 years. Some have set the age limit particularly low at 45 [14] or 50 years [16,18,20], others at 60 years of age [13,19]. One Chinese study with 25,285 participants examined only very old people (≥80 years) [21]. It also seems that there is a dearth of studies in Western countries. The reasons for this lack of research are not known, given the concerns regarding cognitive decline in the elderly worldwide and the aging of the global population in those countries. Supposedly, in Western countries researchers do not divide the population into rural and urban residents, due to the universal health coverage, the easiness in transportation, etc. However, there may still be important differences between rural and urban locations, other than accessibility to health services, that should be taken into account by researchers. Finally, it has been suggested that rural-urban division may be only a rough proxy of the built and social environment in community, and that more specific contextual measurements, such as safety, ethnicity structure, public open space, food environment and local services need to be included if we are to disentangle the effects of place of residence on cognitive health [22].

### 4.5. Implications for Care and Future Research

The findings of the present review are relevant, given the marked differences in demographic and socioeconomic characteristics, lifestyle, medical resources and information provision between urban and rural settings [29]. Moreover, people in urban regions have higher education levels, which is a favorable prognostic factor for MCI and dementia progression [30]. Notably, other chronic diseases associated with cognitive decline and dementia, such as depression, cerebrovascular disease, diabetes and hypertension, may be more prevalent in rural areas compared with urban ones [31,32].

It seems that there may be clinically relevant differences in onset, course and prognosis of MCI with regards to the place of residence (urban vs rural). Those differences should be taken into account for the design of health policies and service delivery in different settings. Health disparities between rural and urban areas are well documented [33] and may undermine the timely diagnosis and optimal management of MCI in the rural context. Moreover, guidelines regarding the management of patients with MCI should be adjusted accordingly to meet the needs of rural residents. Future dementia prevention strategies should identify MCI in rural areas and pay attention to early-stage memory changes in older individuals [34]. Such strategies should include nutritional interventions for rural-dwelling older adults [15] and diabetes prevention in urban areas [30]. Indeed, it has been suggested that health promotion programs and policies against cognitive impairment in the elderly should be tailored and modified according to their place of residence [30]. Prevention should also involve initiations to reduce socioeconomic adversities in rural areas, as well as addressing the childhood adverse experiences that would impact cognitive function in middle-aged and older adults [14]. Notably, it has been recently suggested that rural-dwelling older women should be considered a priority for the prevention of dementia and MCI, due to their longer life-expectancy [34].

Finally, further research is needed on MCI with a focus on rural locations for the better understanding of the impact of living environment on the course and prognosis of MCI. Future research should address potential environmental factors that could be implicated in the onset and course of MCI in rural areas and affect prognosis and outcome. Female rural residents should be particularly studied as there is some evidence that they may be more likely to develop MCI, and no specific protective factors have been identified yet. Indeed, research is ongoing and according to a very recent study, in urban locations there may be more opportunities for social participation, and therefore, the environment is beneficial for cognitive function, alongside the higher education of urban residents [35]. Having social relationships, such as contact with friends, is considered as a protective factor for cognition. Further studies should clarify the content of social relationships in this respect [34].

## 5. Conclusions

The latest research suggests that there may be differences in MCI onset, progression and prognosis with regards to the place of residence, alongside the well-established notion that the prevalence of MCI is higher in rural locations. Different environmental factors may have protective or precipitating effects on cognitive decline, according to place of residence. Future studies should address the impact of rurality on cognitive health by elucidating the mechanisms underlying cognitive decline and by investigating the risk factors that may be associated with the rural context. Meanwhile, prevention strategies should be employed, with a focus on rural-dwelling women. Prevention may comprise of the early detection of MCI in rural areas, nutritional interventions, enhancement of social participation and interventions for the elimination of rural-urban disparities in education and socioeconomic status.

## Figures and Tables

**Table 1:** Risk factors for MCI in rural and urban areas (continued on the top of the next page).

Study	Country	Objective	Study Design	Participants	Main Findings	
					Rural	Urban
Srivastana and Muhammad, 2022 [13]	India	To explore the association between food insecurity and cognitive impairment	Cross-sectional	31,464, 60 years or older	Participants with food insecurity in rural areas had higher odds of cognitive impairment	Lower prevalence of cognitive impairment. Participants reported less food insecurity
Zhang & Zhang, 2022 [14]	China	To explore the association of adverse childhood experiences with MCI	Cross-sectional	11,475, 45 years or older	Family socioeconomic status, food deprivation, neighborhood environment and social relations were associated with MCI	Lower prevalence of MCI. Only father’s education and social relations were associated with MCI
Zhang et al., 2021 [15]	China	To investigate the association between BMI and cognitive impairment in urban and rural areas	Cross-sectional	8221, 65 years or older	Being overweight was associated with cognitive impairment in ages 80+. Being underweight was associated with higher dementia risk in ages 80+.	Obesity was associated with cognitive impairment in ages 65–69
Luo et al., 2019 [16]	China	To explore the association between productive activities and cognitive decline in rural and urban residents	Cross-sectional	13,596, 50 years or older	Paid employment was most beneficial for men. None of the studied productive activities was significantly associated with cognitive decline in women	Lower prevalence of MCI. Caring for grandchildren and volunteering were most beneficial for women
Butcher et al., 2018 [17]	France	To study the differences in plasma CCL11 (eotaxin-1) and cognitive status between rural and urban residents	Cross-sectional	833, 65 years or older	Increased CCL11 was associated with poorer cognitive performance	No association of CCL11 with cognitive performance
Vintimilla et al., 2018 [18]	USA	To study the relationship between potassium plasma levels and MCI	Cross-sectional	510, 50 years or older	Potassium levels were significantly associated with MCI	Potassium was the only electrolyte that successfully predicted MCI status
Tiraphat, 2018 [19]	Thailand	To study the prevalence and risk factors of cognitive impairment in Thai older population living in urban and rural areas	Cross-sectional	6633, 60 years or older	Higher prevalence of MCI. Poor economic condition was a significant predictor of MCI	Perceived poor health status was a significant predictor of MCI
					Female gender, age, education, and active leisure were significant predictors of MCI in both areas	
Tang et al., 2016 [20]	China	To address risk factors for cognitive impairment in urban and rural population	Cross-sectional	7900, 50 years or older	Cognitive impairment was associated with female gender, exposure to pesticides, history of encephalitis or meningitis and head trauma	Cognitive impairment was associated with lack of physical activities and presence of diabetes mellitus
					Prevalence of MCI did not differ between rural and urban residents	

Note: BMI: Body Mass Index; MCI: Mild Cognitive Impairment.

**Table 2:** Prognosis and mortality of MCI in rural and urban areas.

Study	Country	Objective	Design	Participants	Main Findings	
					Rural	Urban
Li et al., 2021 [21]	China	To explore the association of cognitive impairment with all-cause mortality	Longitudinal study	25,285, 80 years or older	No differences in mortality risk compared with urban population with cognitive impairment	Participants had better cognitive function
Xiang et al., 2018 [22]	China	To study the impact of rural-urban community settings on cognitive decline	Cross-sectional, longitudinal	1709, 65 years or older	Poor cognitive initial status	Cognitive decline rate was faster
Mattos et al., 2017 [23]	USA	To compare MCI symptom severity among older rural and urban Appalachians	Cross-sectional	289, 65 years or older	Patients presented for evaluation earlier since symptoms’ onset	Longer time lapse from symptom identification to diagnosis

Note: MCI: mild cognitive impairment.

## Data Availability

Not applicable.
